# Stress Wave Isolation by Purely Mechanical Topological Phononic Crystals

**DOI:** 10.1038/srep30662

**Published:** 2016-08-01

**Authors:** Rajesh Chaunsali, Feng Li, Jinkyu Yang

**Affiliations:** 1 Aeronautics and Astronautics, University of Washington, Seattle, WA, 98195-2400, USA; 2 Department of Physics, South China University of Technology, Guangzhou, 510640, China

## Abstract

We present an active, purely mechanical stress wave isolator that consists of short cylindrical particles arranged in a helical architecture. This phononic structure allows us to change inter-particle stiffness dynamically by controlling the contact angles of the cylinders. We use *torsional* travelling waves to control the contact angles, thereby imposing a desired spatio-temporal stiffness variation to the phononic crystal along the *longitudinal* direction. Such torsional excitation is a form of parametric pumping in the system, which results in the breakage of the time-reversal symmetry. We report that, in quasi-static sense, the system shows topologically non-trivial band-gaps. However, in a dynamic regime where the pumping effect is significant, these band-gaps become asymmetric with respect to the frequency and wavenumber domains in the dispersion relationship. By using numerical simulations, we show that such asymmetry has a direct correspondence to the topological invariant, i.e., Chern number, of the system. We propose that this asymmetry, accompanied by selective inter-band transition, can be utilized for directional isolation of the stress wave propagating along the phononic crystal.

A wide variety of researchers have been striving to attain the ability to control the flow of energy in media for centuries. In particular, one-way transmission of electrical energy by semiconductor diodes and transistors has revolutionized the modern era. On the similar lines, one-way propagation of light, heat, and sound have captured the attention of the scientific community, leading to the invention of novel devices, namely photonic^1^, thermal^2^, and acoustic diodes^3^, respectively. With regard to controlling the mechanical waves in structures, however, researchers constantly face challenges mainly due to the complexity stemming from different modes of stress waves that can simultaneously propagate in the medium^4^. Recently, the advent of phononic crystals and mechanical meta-materials has opened the possibility of controlling such versatile wave propagation in solids^5^,^6^.

One of the most common ways to realize one way transmission in a mechanical system is to break the time-reversal symmetry^7^. Such a non-reciprocal system can also be termed as an isolator in one-dimensional settings. The non-reciprocity can be achieved, for example, by applying an external bias/field^8^ or by introducing non-linearity^9^. In the scenarios when *in-situ* tunability of wave transmission direction is desired, a linear medium would take the precedence over a non-linear one. This is because the allowed direction of wave propagation can be reversed simply by flipping the external bias, which is not the case with *passive* settings of non-linearity-based isolators. Moreover, the isolation characteristics could be tuned by adjusting the magnitude of the external bias directly, thereby making such systems *active*. One could use different types of biases in these active systems. For example, in an acoustic domain, external moving fluid can serve as a bias^10^,^11^. However, non-fluidic structural settings require an external inertial, optical, magnetic, electric or thermal field^12^,^13^,^14^,^15^. These external fields can then couple with the elastic properties of the host structure in order to introduce time modulation of elastic parameters. For the structures that cannot afford aforementioned couplings or are required to be placed in the settings where the application of external non-mechanical fields is not a viable option, it is desired that a purely mechanical system is devised in order to isolate the stress waves.

In this study, we propose a one-dimensional phononic crystal that consists of a chain of cylinders, to achieve the stress wave isolation in a purely mechanical manner. The array of cylinders has shown extraordinary tunability in terms of tailoring the stress wave propagation through them^16^,^17^,^18^. This tunability stems from the Hertz contact law^19^ between adjacent cylinders, where their contact stiffness can be easily varied by changing the contact angle of the cylinders^20^. With this fundamental ability, we explore versatile, one-directional stress wave propagation in the helicoidal phononic crystal. This structure allows both longitudinal and torsional modes of wave propagation in the medium. Remarkably, we use the complex interaction of these two different wave modes to our advantage, and dynamically tune the system’s stiffness for the *longitudinal* wave by having a direct control over the *torsional* modes. In particular, we use a torsional *travelling* wave to induce a specific spatio-temporal modulation of the system’s stiffness, which resembles the sliding lattice potential of the topological Thouless pump in quantum mechanics^21^. This, in turn, makes the system behave as a topological mechanical pump, and thus breaks the time-reversal symmetry. In comparison with the symmetry breakage in conventional topological acoustic22–24 and mechanical25,26 meta-materials in two-dimensions, which results in a robust one-way propagation of edge mode, our time-dependent one-dimensional system results in asymmetric *bulk* wave propagation along the phononic crystal. In this study, we show the topological invariants that we calculate for our one-dimensional system quantify the amount of asymmetry in the dispersion of such bulk waves, in contrast to a two-dimensional system in which those invariants tend to be related to the number of edge modes.

Inspired by the mechanics of the Hertzian contact, the architecture of the helix, and the physics of the Thouless pump, this study claims three-fold novelty as following. First, in terms of design, we propose a purely mechanical system that utilizes a strong coupling between two mechanical wave modes to break the time-reversal symmetry. This implies the control of one mechanical wave mode via another, without resorting to external bias/field. Second, in terms of the fundamental physics of a time-dependent, one-dimensional mechanical system, we show that this system is topologically non-trivial and can be characterized by the topological invariant in two-dimensions, i.e., Chern number^27^. Its non-zero value remarkably acts as a bridge connecting quasi-static and dynamic scenarios, and quantifies the asymmetry observed in dispersion curves. The third novelty is in terms of the mechanism that facilitates the controllable, directional wave propagation, as a consequence of the asymmetry in dispersion curves. To the best of the authors’ knowledge, such type of mechanism – reminiscent of inter-band transition in electronics^28^ and photonics^29^ – has not be observed previously in a purely mechanical system.

## Theoretical Background

### Phononic crystal architecture

**Figure 1:**
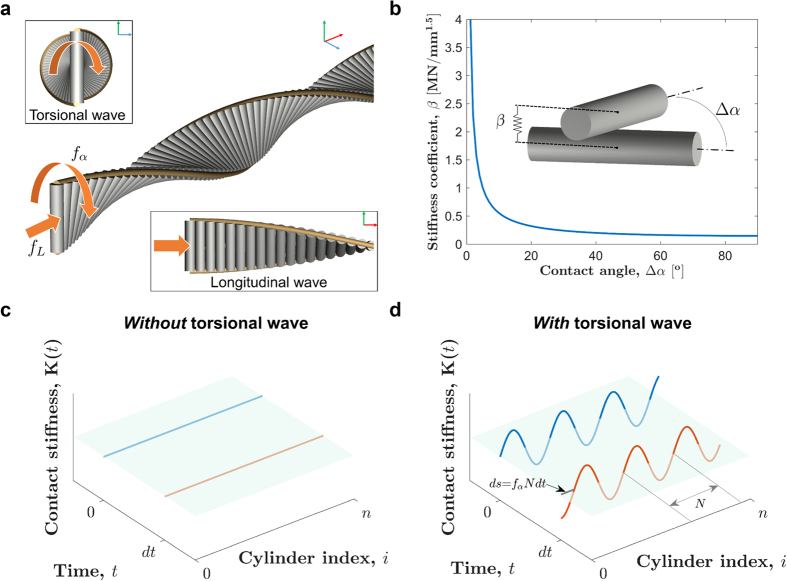
Phononic crystal model. (**a**) Cylinders stacked helically to form a one-dimensional chain. Insets show the two types of wave modes the structure supports. *f*_*L*_ and *f*_*α*_ are the frequencies of longitudinal and torsional travelling waves, respectively. (**b**) Contact stiffness dependence on the angle between two cylinders (Δ*α*), which is perturbed by the torsional wave. (**c**) Uniform contact stiffness for the longitudinal wave propagation in the system *without* torsional excitation. (**d**) Spatio-temporal variation of the same stiffness *with* torsional excitation. The spatial distribution of stiffness moves by *ds* distance in time *dt*.

The phononic crystal to be investigated in this study consists of short cylindrical particles stacked helically as a one-dimensional chain (Fig. 1a). Each cylinder is oriented in a way that the contact angle with its neighbouring particle is uniform throughout the chain at equilibrium. Under the application of compression to the chain, the contact stiffness among the particles in the longitudinal direction is governed by the Hertzian contact law^19^ (see Fig. 1b for the contact between two neighbouring cylinders). When there are no dynamic perturbations of the torsional angles, all cylinders will maintain constant contact stiffness over time (Fig. 1c). However, if a torsional travelling wave is sent from one end of the chain, we would expect it to perturb the contact angles from the equilibrium. The perturbation in the contact angle will, in turn, change the contact stiffness following the relationship in Fig. 1b. This will eventually cause a spatio-temporal modulation of the contact stiffness along the longitudinal direction of the medium (Fig. 1d). Note that this spatio-temporal modulation can be also achieved in an architecture other than the helical array, though we employ this helical structure for the sake of simplicity in this study.

Based on the previous work by Li *et al*.^18^, we establish a framework to define and calculate instantaneous contact stiffness, which governs the wave propagation in the system. For a chain of cylinders, we write the absolute orientation angle of *i*-th cylinder as


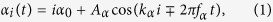


where *α*_0_ is the contact angle between any two cylinders at equilibrium; *A*_*α*_ is the dynamic perturbation in the contact angle caused by a travelling torsional wave; *k*_*α*_ = 2*π*/*N* represents a wavenumber with *N* being the spatial period of the contact angle variation; *f*_*α*_ denotes the frequency of the travelling torsional wave; and 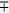 signs indicate *forward* and *backward* direction of the travelling torsional wave, respectively.

We can define the contact angle, i.e., relative angle between two cylinders, as Δ*α*_*i*_(*t*) = *α*_*i*+ 1_(*t*) − *α*_*i*_(*t*). Using the Hertz’s contact law, instantaneous contact force between *i*-th and (*i* + 1)-th cylinders would be *β*_*i*_(*t*)*δ*^3/2^, with *δ* as the relative longitudinal displacement between two cylinders. The time-dependent stiffness coefficient *β*_*i*_(*t*) is of the following form^19^,^20^:


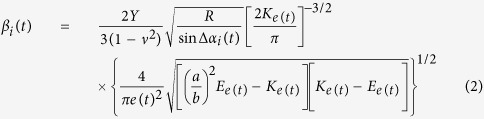


Here, *Y, μ* and *R* denote Young’s modulus, Poisson’s ratio, and the radius of a cylinder. *K*_*e*(*t*)_ and *E*_*e*(*t*)_ represent the complete elliptical integrals of the first and second kinds, respectively. The elliptical contact area has eccentricity 
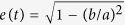
, with *a* and *b* as its semi-major and semi-minor axes, respectively. *b*/*a* is equal to [(1 + cos Δ*α*_*i*_(*t*))/(1 − cos Δ*α*_*i*_(*t*))]^−2/3^, approximately^19^.

### Equations of motion

Once we have formulated the instantaneous contact stiffness between constituents elements, we can now write the equations of motion for the system consisting of *n* cylinders of equal masses *m*. We fix the longitudinal motion and keep the torsional motion free at both ends of the chain. Let *x*_*i*_(*t*) represent instantaneous longitudinal displacement of the *i*-th cylinder, and *δ*_*i*_ be the initial deformation between *i*-th and (*i* + 1)-th cylinders under static pre-compression. For a generic scenario, under the excitation forces *p*_1_(*t*) and *p*_*n*_(*t*) applied to the first and last cylinders of the chain, respectively, we write-


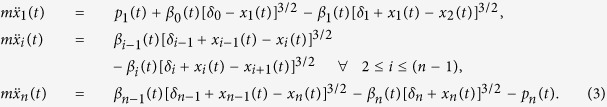


For small instantaneous displacements 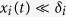, we linearise Eq. (3) and define linear stiffness 
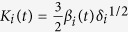
 to obtain the following matrix form-


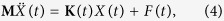


where displacement vector *X*(*t*), stiffness matrix **K**(*t*), mass matrix **M**, and forcing vector *F*(*t*) are expressed as-


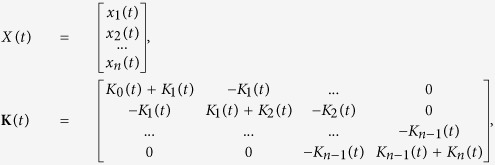



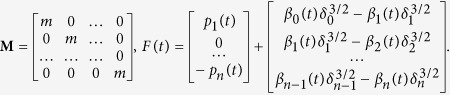


Let us now look at the Eq. (4) – which includes multiple time-dependent terms – in more detail. We have a time-dependent stiffness matrix **K**(*t*) and a forcing vector *F*(*t*), which make the system dynamics fairly complex yet interesting. **K**(*t*) imbibes spatio-temporal modulation of contact stiffness in the system, whereas the forcing term *F*(*t*) has an *external* forcing component as well as a component dependent on stiffness modulation. Under no external force *p*(*t*) applied to the system, we can easily deduce that a travelling torsional wave as in Eq. (1) would enforce time periodicity on these terms. Therefore, we have **K**(*t*) = **K**(*t* + *T*) and *F*(*t*) = *F*(*t* + *T*), where *T* = 1/*f*_*α*_. This periodic evolution of **K**(*t*) represents *parametric pumping* in the system caused by torsional wave. The mechanism is analogous to the pumping action performed, for example, when one wants to change the amplitude of oscillation of a swing without exerting an external force. Instead, one changes the center-of-mass and thus vary the effective swing length with time^30^. **K**(*t*) acts as such a time varying parameter in this case, which is pumped with frequency *f*_*α*_ of the torsional wave.

Note that this parametric pumping has direct consequences on the vibrational modes the system supports. If the perturbation in contact angles were to depend solely on space but not time, i.e., *f*_*α*_ = 0, we see that the second component of *F*(*t*) vanishes, and this equation reduces to the familiar standard form: 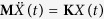 with time *independent* **K**. We can easily analyse it further to obtain eigen-frequencies and Bloch states. However, due to a non-zero *f*_*α*_, simultaneous space-time stiffness modulation is enforced, and a *time-periodic* set of equations with a period *T* represents our system. This period is dictated by the *pumping* frequency *f*_*α*_ of the torsional wave. In this way, our system not only supports the Bloch states caused by spatial variation of parameters, but also allows us to tailor them by an additional degree of freedom, i.e., time, hence taking us to the realm of Floquet-Bloch engineering^31^.

## Results and Discussion

### Quasi-static analysis: Non-trivial topological band-gaps

To understand the system dynamics, we start with analysing the normal modes of vibration in the system. We would take the homogenized form of Eq. (4) by suppressing the forcing term such that-


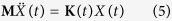


where **K**(*t* + *T*) = **K**(*t*). This time-periodic system of equation resembles the well studied Mathieu’s equation, and it can be solved using Floquet theory^30^. However, we take a different approach to connect our system to an *adiabatic* topological quantum pump^21^. We first analyse the system in *quasi-static* scenario, and then extend the key characteristics to explain *dynamic* scenario of pumping. With regard to quasi-static evolution over time, we assume that the variation in the stiffness matrix **K**(*t*) with respect to time is significantly small, such that the natural modes of vibration can be calculated directly from the matrix at any time instant. Natural frequencies corresponding to such quasi-static evolution of system are named as *static* frequencies^30^. It is worth emphasizing again that *k*_*α*_ in Eq. (1), which is the measure of spatial variation in stiffness, is responsible for frequency band-gaps^18^. On the other hand, the term *f*_*α*_, i.e., the pumping frequency, causes temporal variations in stiffness, and is the measure of how quickly static frequencies are varied over time. If we understand how static frequencies evolve over time, that would give us direct insights into the actual system dynamics for quasi-static pumping. It would also be useful to decipher the complex dynamics in case of rapid pumping (with high torsional wave frequency) where secondary mode of vibration with frequency offsets, namely frequency cascades^32^, can no more be neglected (to be discussed in the later sections).

Under the quasi-static assumption, we can convert Eq. (5) to an eigenvalue problem by substituting time-periodic ansätz 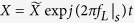 and deduce-


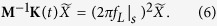


Here *f*_*L*_|_*s*_ denotes the *static* frequency of the *longitudinal* waves, which should not be confused with pumping frequency *f*_*α*_ of the *torsional* waves. For the analysis, we choose *α*_0_ = 10°, *A*_*α*_ = 2°, *N* = 6, and *n* = 300. We take geometrical dimensions and material properties of quartz cylinders from our previous experimental studies (*Y* = 72 GPa, *ν* = 0.17, density *ρ* = 2187 kg/m^3^, height *h* = 18 mm and *R* = 9 mm)^16^. We apply a pre-compression of 40 N to the chain and solve Eq. (6) at each time-step for a small pumping frequency *f*_*α*_ = 0.1 kHz of the *forward* propagating torsional wave.

**Figure 2:**
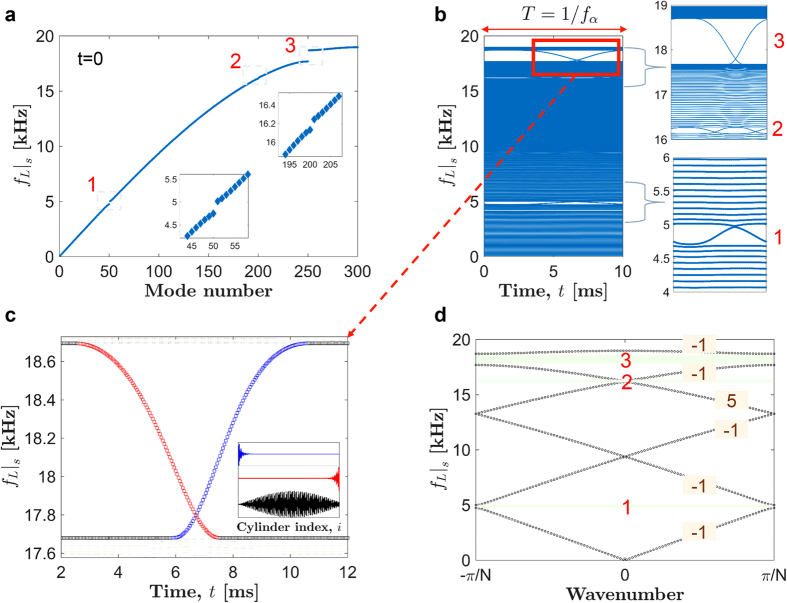
Non-trivial band-topology due to *quasi-static* torsional wave pumping. (**a**) Spatio-temporal variation in stiffness, caused by torsional wave, forces the system’s eigen-frequencies for longitudinal vibration mode to change over time. We plot the eigen-frequencies of a finite size system at one such time instant highlighting the prominent band-gaps: 1, 2, and 3 (insets show zoomed view). (**b**) Evolution of eigen-frequencies over one period of pumping. Insets show close view of frequency evolution inside the band-gaps: 1, 2, and 3. The points of degeneracy (crossing) are noticeable. (**c**) A closer view of the degenerate point inside band-gap 3 showing that edge modes interchange their polarities across the degenerate point, a signature of bulk-boundary correspondence in topological transition. Inset shows the corresponding eigen-modes, both edge and global, represented in different colors. (**d**) Bloch dispersion curve of an infinitely periodic lattice, with Chern numbers assigned to each bands. Non-zero values of gap Chern numbers (summation of Chern numbers of the bands below) confirm the topological nature of all three prominent band-gaps.

In Fig. 2a, we show eigen-frequencies for the longitudinal wave at one time instance, e.g., *t* = 0. In general, we expect five band-gaps in frequency spectrum for the spatial period (*N* = 6) of stiffness variation. However, the perturbation *A*_*α*_ in contact angles is small, and therefore we notice only three prominent band-gaps, labelled as 1, 2, and 3 (insets show zoomed view). We then calculate how these frequencies evolve over time due to the quasi-static pumping of torsional wave. Since the pumping is periodic, we expect evolution of the eigen-frequencies to be periodic as well. In Fig. 2b we show such time evolution for one pumping period *T*. It is important to note that only frequencies on the edges of band-gaps evolve quite distinctly over time. Frequencies corresponding to global modes do not evolve as distinctly as the edge modes. This is intuitive if we rely on the following argument: spatio-temporal stiffness variation in our system is equivalent to a sliding potential, so at each time instant, stiffness variation is just spatially shifted from the one at last time instant. Quasi-statically speaking, this only reflects as a change in the edge modes of a finite lattice, not the global modes. Hence, the modulation is only evident for the modes on the edges. It is also important to note that all three band-gaps evolve distinctly over time with a different number of degeneracies (crossings). We will now shed light on the same in more detail, and establish their non-trivial topological characteristics.

Figure 2c includes a zoomed view of the typical time evolution of modes inside the highest band-gap 3, centred around 18.2 kHz. We observe that the pumping forces the band-gap to open and close periodically over time. Interestingly, such transition is accompanied by a process of phase change for the edge modes across a point of degeneracy. For example, the highlighted mode at the bottom edge of the band-gap transitions from being global to a left localized edge mode, then to a right localized, and global mode again. This implies that the system changes its characteristics with time, as we see edge modes appearing, interchanging their phases, and then disappearing. This time-dependent *bulk-boundary correspondence* indicates breaking for time-reversal symmetry; and it is the classical analogue of the topological band-gaps of the sliding lattice first proposed by Thouless in 1983^21^.

Such non-trivial topological nature of band-gaps manifests the quantization of pumped charge in a time-dependent, one-dimensional quantum mechanical system. The average charge/photon flow in one period of pumping cycle can be quantified using a topological invariant in two dimensions, i.e., Chern number^27^,^33^,^34^. In the current case, we adopt a similar approach to characterize the band-gaps in theoretically possible quasi-static limit. We choose wavevector and time as two independent variables and calculate the Chern number using the method detailed in ref. 25 for a mechanical system. In Fig. 2d, we show the Bloch dispersion curve for an infinitely periodic system. We label each band with the corresponding Chern number. It is known that the topological characteristics of a band-gap is dictated by the “gap Chern number”, which is the summation of the Chern numbers of all the bands below it. In this way, gap Chern numbers −1, 2, and 1 can be associated with three prominent band-gaps: 1, 2, and 3, respectively. It is evident that these non-zero values indicate non-trivial band topology of the system under time evolution. It is also remarkable that these gap Chern numbers directly explain the number of degenerate points during one period of time evolution of band-gaps as seen in Fig. 2b. Opposite signs of the numbers associated with band-gaps 1 and 3 simply denote the opposite phase reversal of the edge modes (interchanging the blue and red colors of Fig. 2c). In the later sections, we would discuss how these topological invariants relate to the asymmetry in the dispersion curve, and hence influence the dynamic response of our system.

### One-way transmission of stress waves

**Figure 3:**
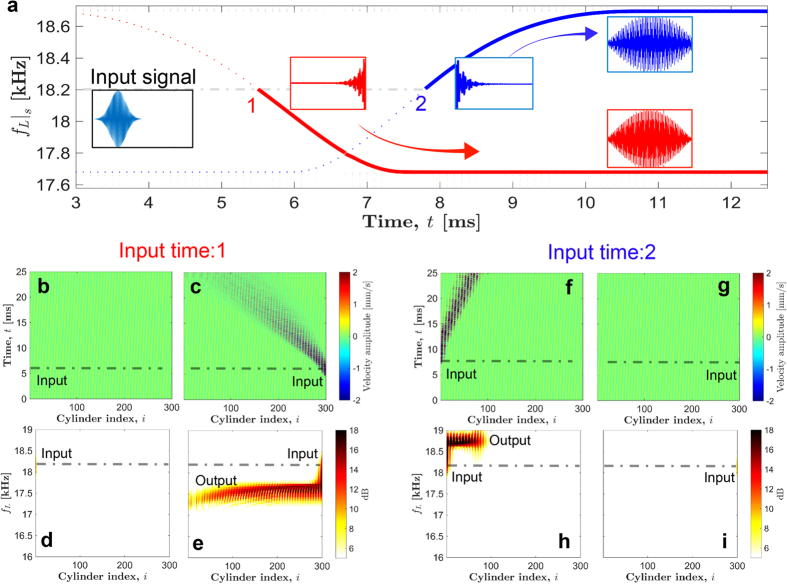
Implication of bulk-boundary correspondence for *small* pumping frequency. (**a**) Typical topological transitions of the eigen-modes inside band-gap 3 as also shown in Fig. 2c. System’s ability to selectively convert a left (and right) localized edge mode to a global mode with higher (and lower) frequency is highlighted at two unique time points. A full numerical simulation is carried out with a Gaussian pulse centred at *f*_*L*_ = 18.2 kHz as an input to the system. (**b**,**c**) Spatio-temporal map of cylinder velocity when the input is from left and right end of the chain, respectively, at time point **1**. (**d**,**e**) Propagating pulse frequency corresponding to the velocity maps. (**f**–**i**) The same plots obtained by sending the pulse at time point **2**.

We now verify the results obtained through quasi-static analysis, and demonstrate the implications of the bulk-boundary correspondence in the system by carrying out full numerical simulations at a *small* pumping frequency, *f*_*α*_ = 0.01 kHz. In Fig. 3a, topological transitions are highlighted for the highest band-gap 3, in which we notice that there are two time instances which are particularly important at a given input frequency of longitudinal wave. The first instance (indicated by Point 1 in Fig. 3a) is a point from which an edge mode – localized at the *right* end – evolves to a global mode. The other instance (Point 2 in Fig. 3a) is where an edge mode – localized at the *left* end – evolves to a global mode. To verify this wave evolution, we send a short pulse of longitudinal wave (specifically, 0.8 ms long Gaussian force excitation at *f*_*L*_ = 18.2 kHz) from both left and right ends of the chain separately and see how the pulse propagates in the medium. We send these pulses at the two different time points 1 and 2, and solve Eq. (3) using the Runge-Kutta scheme for all further numerical simulations. First, when we send pulses at the time point 1, as shown in Fig. 3b,c, we observe that the longitudinal waves can propagate only from the right to the left end. This is accompanied by a frequency shift shown in Fig. 3d,e, where the shifted frequency corresponds to the lower edge of the band-gap. In the reverse direction, we do not see the transmission of the input pulse. In the second case when the pulse is sent at the time point 2, Fig. 3f–i show that the pulse can travel only from the left to the right end. In addition, frequency shifts to a higher value, which corresponds to the upper edge of the band-gap. We also notice that wave speeds are not equal in these cases because of the inherent dispersion in the system, where higher frequency wave propagates slowly. Therefore, it is clear that the system responds selectively to excitation sent from left and right end of the chain, and introduces different frequency shifts in accordance with the bulk-boundary correspondence in quasi-static analysis.

It should be also noted that if we flip the sign of the pumping frequency (essentially of the phase factor 2*πf*_*α*_*t* in Eq. (1)), we can easily see that the above observations would be reversed. For example, the pulse sent from the left end would propagate with lower frequency, and the pulse sent from the right end would propagate with higher frequency. This would also reflect in the change of sign (positive to negative, and vice-versa) of the Chern numbers associated with the bands. This implies that we break the time-reversal symmetry in the system for a non-zero pumping frequency. Therefore we show that our system offers non-reciprocity for a mechanical pulse, and the fundamental mechanism relies on topological bulk-boundary correspondence inside the band-gaps in a quasi-static limit. At this juncture, a natural question to ask would be: how is this non-trivial topology of band-gaps important when we break the quasi-static assumption and start applying high-frequency torsional waves? We note that quasi-static evolution of eigen-frequencies (Fig. 2b) no longer would dictate the dynamics directly. We would have to take dynamic factors into our consideration once the torsional wave has a significantly high frequency. Would the non-reciprocity shown in the quasi-static limit still hold true? How do the topological invariants translate into the dynamic system? We would answer these questions in the following sections.

### Dynamic analysis: Asymmetry in dispersion curves

**Figure 4:**
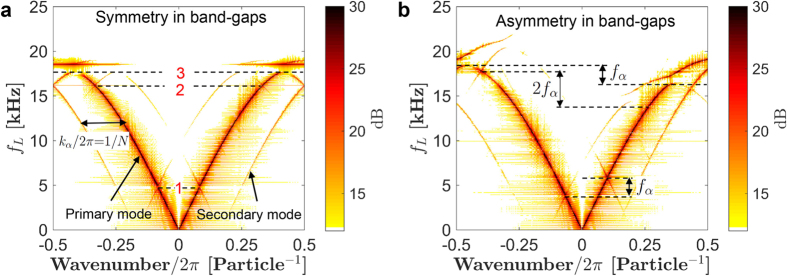
Dispersion curves obtained using full numerical simulations to show the effect of high pumping frequency. (**a**) *f*_*α*_ = 0, i.e., a system with only spatial modulation in stiffness, has a symmetric dispersion curve. The three prominent band-gaps: 1, 2, and 3 are marked. (**b**) Torsional wave (with *f*_*α*_ = 2 kHz in this case) breaks the time-reversal symmetry, and thus results in asymmetric band-gaps for rightward (*positive* wavenumber) and leftward (*negative* wavenumber) propagating longitudinal wave. The frequency offsets for the band-gaps are indicated, and those are dictated by corresponding topological invariant, i.e., the summation of Chern numbers of bands below it, defined in quasi-static sense.

To investigate the dynamic effect of the torsional waves on the longitudinal ones, we solve Eq. (3) computationally for two different torsional wave frequencies: 0 kHz and 2 kHz, while other system parameters are kept the same. The first case represents a chain with only spatial variation of stiffness, whereas the later also incorporates the effect of simultaneous temporal modulation. We give an impulse excitation at one end of the chain to excite all possible vibration modes. By using Fast Fourier Transformation (FFT) of velocity map, we obtain dispersion curves as per extended Brillouin zone scheme. For *zero* pumping frequency, the dispersion curves are perfectly symmetric with respect to the zero longitudinal wavenumber axis (Fig. 4a). In particular, we see symmetric Bloch band-gaps on both wavenumber and frequency domains. Also noticeable are the symmetric secondary mode branches that are horizontally shifted by ±*mk*_*α*_/2*π* with *m* being an integer that represents the interference order as a result of spatial variation of stiffness in the system^18^.

On the other hand, as we introduce a significant pumping frequency (*f*_*α*_ = 2 kHz), time-reversal symmetry is broken, and we notice asymmetry in the dispersion curves (Fig. 4b). Firstly, the secondary mode branches are asymmetric. This asymmetry can, in turn, be quantified as a vertical shift equivalent to ±*lf*_*α*_, where *l* is an integer representing the mode index (primary mode being *l* = 0). However, the most remarkable observation is that each band-gap, on the primary mode branch, shows a unique asymmetry with respect to *positive* and *negative* wavenumber axes. Those are not only distinct in terms of the magnitude of frequency and wavenumber offset but also in their directional shifts. For example, band-gap 1 shifts *up* for positive wavenumbers, whereas band-gap 3, even though showing the same frequency offset, shifts *down* for the positive wavenumbers. Band-gap 2, however, shows entirely distinct offset with double the magnitude compared to those of band-gap 1 and 3.

In order to explain this key observation, we refer to our earlier section on quasi-static analysis. We had shown that the system supports non-reciprocity for mechanical waves based on the bulk-boundary correspondence quantified by a topological invariant in two dimensions. In particular, the degeneracy (mode crossing) – which occurs during the time evolution of eigen-frequencies (Fig. 2b) – essentially influences the system response and results in non-reciprocity with distinct frequency shifts (Fig. 3). In this way, a degenerate point can be treated as the core element in the pumping due to the torsional wave. The *number* and the *type* of degenerate points would, thus, dictate the dynamics *in* and *around* the respective band-gap. As stated earlier, the gap Chern numbers associated with the band-gaps would give us this number and the type of degeneracy. We find that these numbers are −1, 2 and 1 for band-gaps 1, 2, and 3, respectively. Hence, for the pumping frequency of *f*_*α*_, these band-gaps would be individually pumped at the rate of −*f*_*α*_, 2*f*_*α*_ and *f*_*α*_, respectively. This is exactly what is reflected as the distinct asymmetry of band-gaps in the dispersion curve shown in Fig. 4b. Therefore, we find an interesting connection between the asymmetry of band-gaps caused by dynamics pumping, and their topological characteristics in the quasi-static limit. We would like to mention that rapid pumping into the system might cause instability, so we limit ourselves to a finite system analyzed for a finite time. Detailed discussion on instability in such systems can be found in the earlier work by Li *et al*.^18^.

### Non-reciprocity and inter-band transition

**Figure 5:**
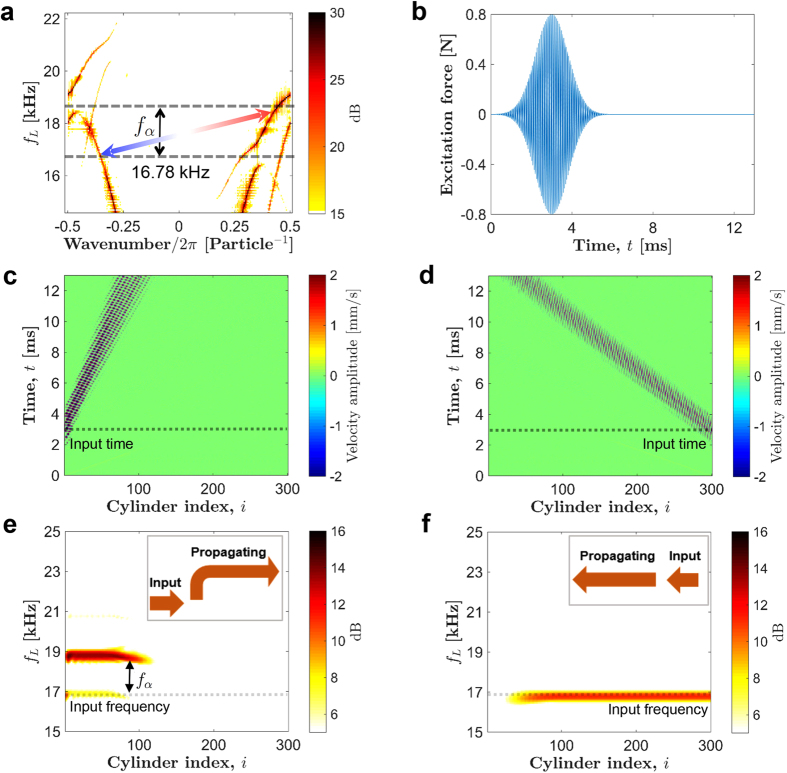
Implication of asymmetric band-gaps. (**a**) A zoomed view of the highest band-gap 3 in the dispersion curve at *f*_*α*_ = 2 kHz. System’s response is examined at input excitation frequency *f*_*α*_ = 16.78 kHz. The arrow tips denote leftward and rightward propagating frequencies indicating inter-band transition. (**b**) Gaussian profile of the input force that is applied at the ends of the cylinder chain. (**c**,**d**) Spatio-temporal map of cylinder velocity when signal is sent from the left (right) end of the chain. (**e**,**f**) The corresponding frequency content of the propagating pulse. We observe that rightward travelling pulse propagates with a higher frequency (slower speed) compared to that of input pulse, however leftward propagating pulse does not see any such frequency shift (insets for summary).

We now explore the implication of these asymmetric band-gaps, and their potential to tailor mechanical wave propagation in the medium. Referring to Fig. 5a, a zoomed view of the same asymmetric dispersion curve at pumping frequency *f*_*α*_ = 2 kHz, we focus on the widest and highest band-gap 3. We choose a longitudinal travelling pulse with frequency *f*_*L*_ = 16.78 kHz, such that it lies inside and outside of the band-gap of the primary mode for the right and left branches of dispersion curve, respectively. We use 0.8 ms long Gaussian pulse (Fig. 5b) with the aforementioned central frequency *f*_*L*_ to give force excitation and send the pulse from both ends of the chain. Figure 5c,d show the spatio-temporal map of particles’ velocities when the system is excited from left and right ends, respectively. Interestingly, we observe that the system allows the injected pulse to propagate in both directions. However, it is remarkable that wave frequency is direction-dependent, which makes the wave speed direction-dependent due to dispersion. We show the spectrum of the propagating wave in Fig. 5e,f. This is obtained by running the numerical simulation for 13 ms, and then doing FFT on the velocity map. It is evident that the wave travelling from left to right has a *higher* frequency compared to the injected one at 16.78 kHz, and the difference is equal to the pumping frequency *f*_*α*_. Note that the stoppage of the highlighted parts in figures does not mean that waves stop propagating at that point. On the other hand, a pulse injected at the right end propagates with the same speed without any frequency shift. We observe even more interesting wave directional behavior in the presence of a defect in the medium (Supplementary Note).

This is indeed a peculiar type of wave dynamics we observe in our system. Though, an input wave can propagate in *both* directions, there is non-reciprocity in terms of the frequency spectrum. Different frequencies propagate in opposite directions. This is in contrast to usual cascading process in time-dependent systems, in which we observe wave mixing effect due to the presence of *multiple* modes separated by the pumping frequency^32^. In the present case, however, we see only two types of modes propagating in the medium instead of multiple ones. We identify that the selection of these two modes can be linked to inter-band transition mechanism^28^,^29^. Inter-band transition is facilitated in a way that a frequency *lower* than a band-gap region propagates in one direction (left), and a frequency *higher* than the band-gap region propagates in the opposite direction (right). These are indicated as the tips of the arrow marked in Fig. 5a. Clearly, those will have different wavenumbers as well. The difference in frequencies would be equal to the offset in band-gaps. As we discussed previously, topological invariants (gap Chern number) dictate this offset, which is *f*_*α*_ for the current band-gap. Hence, we see the modes travelling in opposite directions lie on different bands of the dispersion curve (one lower than gap, and one higher); and hence the term *inter-band* transition is justified.

## Summary

In this study we have proposed a purely mechanical device based on a helical phononic crystal, which shows non-reciprocity in terms of stress wave propagation. We used the coupling of two mechanical waves – longitudinal and torsional modes – to achieve the breakage of time-reversal symmetry. We related such symmetry breaking to the topological nature of band-gaps induced by spatio-temporal variation of system parameters. In this phononic crystal, the topological characteristics represented by the Chern number directly dictate the level of asymmetry associated with each band-gap in the dispersion curves. Notably, the asymmetry in the dispersion curve can be used to achieve the inter-band transition at a specific frequency range. Towards the practical significance, if the higher frequency mode in such asymmetric wave propagation can be filtered, e.g., by using the fact that it would attenuate more in the presence of material dissipation or by taking advantage of its slower wave propagation speed, the current system would serve the purpose of stress wave isolator, allowing stress waves only in one-direction. Moreover, the allowable direction as well as general characteristics of the longitudinal wave can be simply controlled by fine-tuning the torsional wave parameters, thereby making the proposed system active and tunable.

## Additional Information

**How to cite this article**: Chaunsali, R. *et al*. Stress Wave Isolation by Purely Mechanical Topological Phononic Crystals. *Sci. Rep.* **6**, 30662; doi: 10.1038/srep30662 (2016).

## Supplementary Material

Supplementary Information
