# Supplementary material for: Stress Wave Isolation by Purely Mechanical Topological Phononic Crystals
Source: Sci Rep. 2016 Aug 1;6:30662. doi: 10.1038/srep30662 (PMC4967924; doi:10.1038/srep30662)
Supplement: Supplementary Information [file srep30662-s1.pdf]

Stress Wave Isolation by Purely Mechanical Topological Phononic Crystals

Rajesh Chaunsali[1], Feng Li[1,2], and Jinkyu Yang[1,*]

[1]Aeronautics and Astronautics, University of Washington, Seattle, WA, USA, 98195-2400
[2]Department of Physics, South China University of Technology, Guangzhou, China, 510640
[*]jkyang@aa.washington.edu

Supplementary data: Scattering from large defects

In this section, we evaluate the effect of large defects on the non-reciprocal behavior of the current system. As a preliminary analysis, we consider a mass defect which is assigned by doubling the mass of one cylinder at the center of the chain. We keep rest of the parameters the same. We send a Gaussian pulse at 16.78 kHz from both ends of the chain and see how the propagating waves interact with the defect. We would expect that the defect would scatter a fraction of energy in the form of reflected waves; and the rest would be simply transmitted to the other side of the defect. In Fig. 1a, we show the spectrum of propagating pulse injected from left end of the chain. As discussed in the main manuscript, we first see a frequency shift to a higher value as soon as the input excitation enters the system. Wave is then partially reflected and transmitted due to the defect. However, what is interesting is that reflected wave again is at the original input frequency, and that is because the system can allow the wave to propagate in backward direction only at the input frequency (see the main text). On the contrary, when the chain is excited from the right end, Fig. 1b shows how the wave first travels in the same frequency. But after the scattering, the reflected wave attains a higher frequency. These interesting wave inter-band transfers in the presence of a defect have been summarized in the insets attached to each plot.

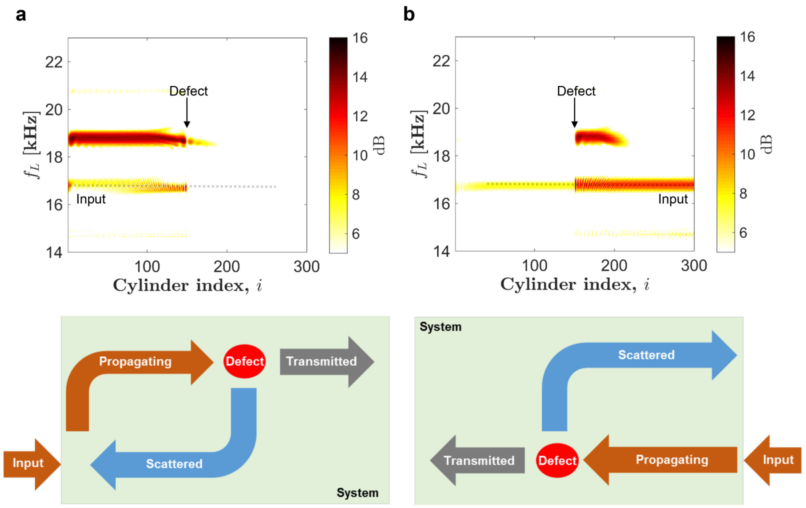

Figure 1. Effect of large mass defect on non-reciprocity in the system: (a)-(b) Frequency content in the traveling pulse as input is given from left (right) end of the chain. Input, propagating, transmitted and scattered frequency components are highlighted using the schematics below.
